# Transcriptome and Metabolome Reveal Key Genes from the Plant Hormone Signal Transduction Pathway Regulating Plant Height and Leaf Size in *Capsicum baccatum*

**DOI:** 10.3390/cells13100827

**Published:** 2024-05-13

**Authors:** Na Xing, Xiaoqi Li, Shuhua Wu, Zhiwei Wang

**Affiliations:** 1 Key Laboratory for Quality Regulation of Tropical Horticultural Crops of Hainan Province, School of Breeding and Multiplication (Sanya Institute of Breeding and Multiplication), Center of Nanfan and High-Efficiency Tropical Agriculture, Hainan University, Sanya 572025, China; xn98123456@163.com (N.X.); 17889848718@163.com (X.L.); 21210902000015@hainanu.edu.cn (S.W.); 2 Key Laboratory for Quality Regulation of Tropical Horticultural Crops of Hainan Province, School of Tropical Agriculture and Forestry, Hainan University, Haikou 570228, China

**Keywords:** pepper, phytohormones, plant height, leaf size, transcriptome

## Abstract

Plant structure-related agronomic traits like plant height and leaf size are critical for growth, development, and crop yield. Defining the types of genes involved in regulating plant structure size is essential for the molecular-assisted breeding of peppers. This research conducted comparative transcriptome analyses using *Capsicum baccatum* germplasm HNUCB0112 and HNUCB0222 and their F_2_ generation as materials. A total of 6574 differentially expressed genes (DEGs) were detected, which contain 379 differentially expressed transcription factors, mainly including transcription factor families such as TCP, WRKY, AUX/IAA, and MYB. Seven classes of DEGs were annotated in the plant hormone signal transduction pathway, including indole acetic acid (IAA), gibberellin (GA), cytokinin (CK), abscisic acid (ABA), jasmonic acid (JA), ethylene (ET), and salicylic acid (SA). The 26 modules were obtained by WGCNA analysis, and the MEpink module was positively correlated with plant height and leaf size, and hub genes associated with plant height and leaf size were anticipated. Differential genes were verified by qRT-PCR, which was consistent with the RNA-Seq results, demonstrating the accuracy of the sequencing results. These results enhance our understanding of the developmental regulatory networks governing pepper key traits like plant height and leaf size and offer new information for future research on the pepper plant architecture system.

## 1. Introduction

Pepper (*Capsicum* ssp.) belongs to the genus *Capsicum* in the Solanaceae family and has a long history of cultivation and high economic value. The plant structure-related traits like plant height and leaf size are two critical phenotypic characteristics of dicotyledonous plants and play a vital role in endurance against adverse conditions. In wheat, for example, plant height is a phenotypic character correlated with Fusarium head blight susceptibility [1]. Plant height is an important agronomic trait that influences the plant architecture of a crop [2]. Plant height is an important factors affecting plant type, a quantitative trait controlled by multiple genes with a complex genetic basis. It is regulated by various factors at both the physiological and molecular levels [3,4]. The semi-dwarf genes presented in the Green Revolution efficiently eluded crop lodging and significantly increased crop yields [5,6]. Most of China’s pepper cultivars are tall, prone to falling over, and not appropriate for mechanized production. The selection and use of dwarf pepper structures is one effective measure to overcome the previously mentioned problems. Studies have revealed that dwarfing is an imperative plant structural trait in crop breeding, and most dwarfing genes are responsible for the short structure of the plant by regulating the balance of endogenous plant hormones, which in turn interfere with cell elongation or cell proliferation, ultimately leading to the dwarfing of the plant [7]. The compact and short plant structure can increase planting density and improve crop yield. It also has anti-fall properties, making the plant less susceptible to pathogen attacks, and is suitable for mechanized management and harvesting [8,9]. Phytohormones are a class of endogenous signaling molecules capable of regulating plant growth and development at very low concentrations and play a critical role in plant growth and development. Studies on dwarfing *Arabidopsis* thaliana and rice mutants have found that plant dwarfing is mainly associated with the production of hormones such as auxin, gibberellins, brassinosteroids, cytokinin, and abscisic acid.

Plant organ size varies from species to species, and the precise regulation of organ size is crucial for the structural and functional basis of plant survival and reproduction on Earth [10]. Leaves are an essential part of the plant structure as they play a role in photosynthesis, respiration, light perception, and transpiration and will affect the plant’s yield [11,12,13]. Leaves provide energy for plant growth and development through photosynthesis and respiration. Leaf size is a quantitative trait controlled by multiple genes. Previous studies have revealed that leaf size is determined by cell size and number [14]. Cell growth must be balanced by cell division and expansion to achieve stable tissue growth and the determined leaf morphology [15]. Hence, exploring the mechanism of leaf size regulation can enrich the critical basis of leaf development and provide imperative information regarding leaf size regulation and variety improvement.

To comprehensively understand the underlying mechanism related to the development of peppers, we utilized RNA-seq and LC-MS/MS analysis techniques to study the mechanism of pepper plant structure regulation by endogenous hormones at the molecular level, which provided a reference for revealing the mechanism of pepper plant height and leaf size regulation and plant improvement.

## 2. Materials and Methods

### 2.1. Plant Materials

Two *Capsicum baccatum* germplasm HNUCB0112 (Short plants with small-size leaves) and HNUCB0222 (Long plants with larger-size leaves) were used and abbreviated as CB112 and CB222. The maternal parent CB222 was crossed with paternal parent CB112 at Hainan University in December 2021. In April 2022, F_1_ seeds were obtained and sown further for germination. A plant population with the same phenotype was obtained, further self-crossed, and finally, F_2_ seeds were obtained. Next, the 300 F_2_ generation single plants were used as the test material for the experiment. The sampling process was completed based on the plant height and leaf size, mainly plants with small, extreme dwarf leaves and large, extremely tall leaves were selected as materials, and named the Df2 and Hf2. Notably, the fully developed leaves were chosen for the sequencing samples and 12 sequencing samples were collected, each with three biological replicates.

### 2.2. Plant Height and Leaf Size Data Measurements

The vertical distance in cm from the start of the ground to the highest point of the plant was measured for CB112, CB222, Df2, and Hf2 in the natural growth state during the ripening of the peppers. At the onset of flowering, the length from the leaf base to the leaf apex and the width at the widest point of the largest normally growing leaf in the plant were measured in cm. Statistical analysis was performed by calculating the mean and standard deviation, and a one-way analysis of variance (ANOVA) was performed to analyze the significant differences (for *p* < 0.001, *p* < 0.01 and < 0.05).

### 2.3. Scanning Electron Microscope Observation and Analysis

Mature leaves of CB112 and CB222 were collected for microscopic analysis and scanning electron microscope (SEM) observation. To determine the size of the epidermal cells, square slices measuring 0.5 cm × 0.5 cm were cut from the middle of the leaf. The surface stains of the sample were removed through gentle rinsing with Phosphate-buffered saline (PBS). The tissue samples were promptly placed in the electron microscope fixative upon extraction and subsequently fixed at room temperature while shielded from light for 2 h. They were then relocated to a storage environment at 4 °C. Wash tissue blocks with 0.1 M phosphate buffer (PB) were washed three times, 15 min each. Then, transfer tissue blocks were placed into 1% OsO4 in 0.1 M PB for 1–2 h at room temperature. After that, wash tissue blocks in 0.1 M PB were washed three times, 15 min each. Dehydration occurred as follows: 30% ethanol for 15 min, 50% ethanol for 15 min, 70% ethanol for 15 min, 80% ethanol for 15 min, 90% ethanol for 15 min, 95% ethanol for 15 min, two changes of 100% ethanol for 15 min, and, finally, isoamyl acetate for 15 min. Samples were dried with a critical point dryer (K850, Quorum, London, UK). The sample was placed on conductive carbon film double-sided tape and placed on the sample stage of the ion sputterer (MC1000, Hitachi, Tokyo, Japan) for gold spraying for 30 s. The specimen was later observed and photographed using a scanning electron microscope (SU8100, Hitachi, Tokyo, Japan). Each intact cell and stomata was counted as 1, and those with fewer than one cell or stoma near the edge of the visual field were always counted as 0.5. Three replicates each of CB112 and CB222 were created.

### 2.4. Comparative Cytological Analysis

The main stem tissues of CB112 and CB222 were taken from the middle part of the fourth internode of the main stem at a thickness of 0.5 cm, and the cross-sectioned leaf tissues of the mature leaves near the middle part. We placed them in FAA fixative. Paraffin slides were dewaxed as follows: two changes of environmentally friendly dewaxing transparent liquid for 20 min, two changes of pure ethanol for 5 min, 75% ethanol for 5 min, and the slides were kept in tap water. We placed sections into safranin O staining solution for 2 h and washed with tap water to remove excess dye. Decolorization: we placed slides into 50%, 70%, and 80% alcohol for 3–8 s. Fixed green staining: we placed sections into plant solid green staining solution for 6–20 s, followed by anhydrous ethanol three-cylinder dehydration. Transparent and sealing: we placed sections into three cylinders of xylene for 5 min. Finally, we mounted the tissue section with neutral gum. We observed the sample under a microscope (Nikon Eclipse E100, Nikon, Tokyo, Japan), took images, and analyzed.

### 2.5. Plant Hormone Testing

Extraction and detection of hormones: tissue samples were taken from the middle of the fourth internode of the main stems of CB112 and CB222, named S112 and S222, and samples of mature leaves, named L112 and L222, of which there were three biological replicates, were used for each sample. After the sample was pulverized using liquid nitrogen, 1.0 g was measured and placed in a 15 mL centrifuge tube. Subsequently, 5 mL of extraction solution (80% methanol–water solution with 0.5% formic acid) was added, soaked at 4 °C overnight, extracted using ultrasonic shock, and centrifuged. The supernatant was collected, and 3 mL of the extraction solution was added for further extraction. The combined supernatant was then left to settle, and 6 mL of the extract was accurately taken out and diluted five-fold with distilled water for purification. The MCX solid phase extraction column was activated and equilibrated with 5 mL of methanol and 5 mL of water. Subsequently, the liquid was passed through the column, eluted with 10 mL of 5% ammoniated methanol after being drenched with 6 mL of 2% formic acid in water, and the eluate was collected. The eluate was evaporated to near dryness using nitrogen, reconstituted with 1 mL of 20% aqueous methanol, centrifuged, and the resulting supernatant was transferred into a brown sample bottle and placed in the machine. Mass spectrometry conditions were as follows: the Electrospray Ionization Source (ESI) operated in both positive and negative ion ionization modes, with an Ion Source Temperature of 500 °C, Ion Source Voltages of 5500 V/4500 V, Curtain Gas at 30 psi, and Atomizing Gas and Auxiliary Gas both at 50 psi. The ion pairs utilized for quantitative analysis are presented in Table S11. All data were processed using the AB Analyst chemical workstation. The mixed standard solution underwent stepwise dilution to produce a series of standard solutions, and the standard curve was plotted with the standard concentration x (ng/mL) as the horizontal coordinate and the peak area value of the control y as the vertical coordinate to calculate the content of the target in the samples. Data analysis was performed as follows: a one-way analysis of variance (ANOVA) was performed to analyze the significant differences (for *p* < 0.01 and < 0.05).

### 2.6. Metabolomic Analysis

The sample collection, metabolite extraction, LC-MS/MS uptake, and qualitative and quantitative metabolite analysis were performed by Novogene Technology Co. (Beijing, China). Firstly, the raw files obtained by mass spectrometry were imported into Compound Discoverer 3.3 software for spectral processing and database searching to acquire the qualitative and quantitative results of the metabolites, and then the data were subjected to quality control procedures to verify the accuracy and reliability of the results. The primary databases for the functional and taxonomic annotation of identified metabolites comprise KEGG, HMDB, LIPID MAPS, and others. A comprehensive understanding of the functional properties and classification of various metabolites can be achieved through the utilization of these databases for annotating the identified metabolites. Thresholds were set at VIP > 1.0, FC > 1.2, or FC < 0.833, and a *p*-value < 0.05 [16,17,18]. The identified metabolites meeting these criteria were considered as differential metabolites.

### 2.7. Transcriptome Analysis

The transcriptome sequencing was conducted by Novogene (Beijing, China). RNA was extracted from pepper leaf tissues using standard extraction methods, followed by rigorous quality control of the RNA samples. The integrity of the RNA samples was primarily evaluated using an Agilent 2100 bioanalyzer (Agilent Technologies, Santa Clara, CA, USA) to ensure accurate detection of RNA integrity. The mRNA libraries from each sample were sequenced using Illumina, San Diego, CA, USA (Novaseq-PE150). In order to ensure the quality and dependability of data analysis, the raw data need to be filtered to remove reads with connectors (adapters), to remove reads containing N, and to remove low-quality reads [19]. After filtering the raw data, checking the sequencing error rate, and checking the distribution of GC content, the clean reads necessary for subsequent analysis were obtained. HISAT2 2.2.1 software was used to quickly and precisely compare the clean reads with the reference genome and obtain the localization information of the reads on the reference genome [20]. New transcript assembly was performed using StringTie 2.1.4 software. Differential analysis was performed using DESeq2 4.1.1 software [21], and genes were considered differentially expressed when they had |log2(Fold Change)| ≥ 1 and padj ≤ 0.05 and were subjected to GO and KEGG analysis.

### 2.8. WGCNA Analysis

The co-expression network was analyzed using the WGCNA in R (Version 3.5.0) package, and the soft threshold (R^2^ > 0.8) was selected based on the FPKM values of all genes. Hierarchical clustering was performed on the genes within the network to create a hierarchical clustering tree, and then the tree was cut into different modules using dynamic shearing to merge similar modules. Co-expression networks were visualized using Cytoscape V.3.7.1 software.

### 2.9. qRT-PCR Analysis

To validate the precision of the transcriptome data, qRT-PCR was conducted. The key genes related to the growth and development of peppers were selected among the DEGs for fluorescence quantitative PCR verification. Total RNA was extracted with the RNA extraction kit, and RNA was reverse transcribed to cDNA. Primer design was conducted using the utilization software Primer 5.0, and validation was performed with Oligo 7 and Primer-BLAST. Real-time quantitative PCR was performed on a fluorescent quantitative PCR instrument using SYBR^®^ Premix Ex Taq II (2×) (Vazyme, Nanjing, China). Actin was used as the internal reference gene. All samples were amplified in three biological and technical replicates. The data were analyzed by qRT-PCR using the ΔΔCt method for reference genes.

## 3. Results

### 3.1. Phenotypic Analysis of Two Pepper Cultivars

#### 3.1.1. Phenotypic Analysis of Plant Height

**Figure 1:**
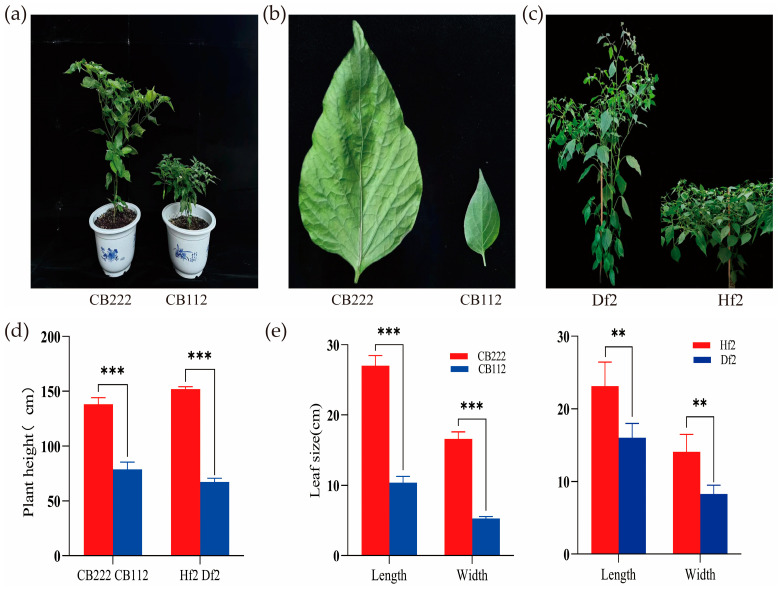
(**a**) Phenotypic characteristics of the two pepper varieties (CB112 and CB222); (**b**) leaf morphology; (**c**) F_2_ generation plant comparison; (**d**) plant height difference between CB112 and CB222, and plant height difference between Df2 and Hf2; (**e**) leaf size difference between CB112 and CB222 and leaf size difference between Df2 and Hf2. (** denotes *p* < 0.01, *** denotes *p* < 0.001.)

In this study, the phenotypic characteristics of CB112 and CB222 significantly differed regarding growth and development (Figure 1a). During the flowering and fruiting stage, the varieties CB112 and CB222 had 5 and 13 primary stem nodes, respectively, and plant heights of 78.9 ± 6.5 cm and 138 ± 6.1 cm, respectively, with highly significant differences (Figure 1d). CB112 was utilized as the male parent and CB222 as the female parent to obtain the F_1_. In the F_2_ population, plant height and leaf size traits were segregated, with two distinct characteristics: tall plants with large leaves and dwarf plants with small leaves. The height of the dwarf pepper plants was 67.4 ± 2.2 cm, and the tall pepper plants were 151 ± 2.2 cm (Figure 1c).

#### 3.1.2. Phenotypic Analysis of Leaf Size

The leaf blades of CB112 and CB222 were significantly different in morphological characteristics. Leaf length, leaf width, and leaf area were 10.4 ± 0.9 cm, 5.3 ± 0.3 cm, and 38.5 ± 5.3 cm^2^ for CB112, and 27.0 ± 1.5 cm, 16.6 ± 0.9 cm, and 314.79 ± 33.5 cm^2^ for CB222, respectively (Figure 1b). In the F_2_ population, the leaf length, leaf width, and leaf area were 16.0 ± 2.0 cm, 8.3 ± 1.2 cm and 100.6 ± 24.1 cm^2^ for dwarf peppers and 23.1 ± 3.3 cm, 14.1 ± 2.4 cm and 249.3 ± 76.7 cm^2^ for taller peppers, respectively. In both cases differences were highly significant (Figure 1e).

**Figure 2:**
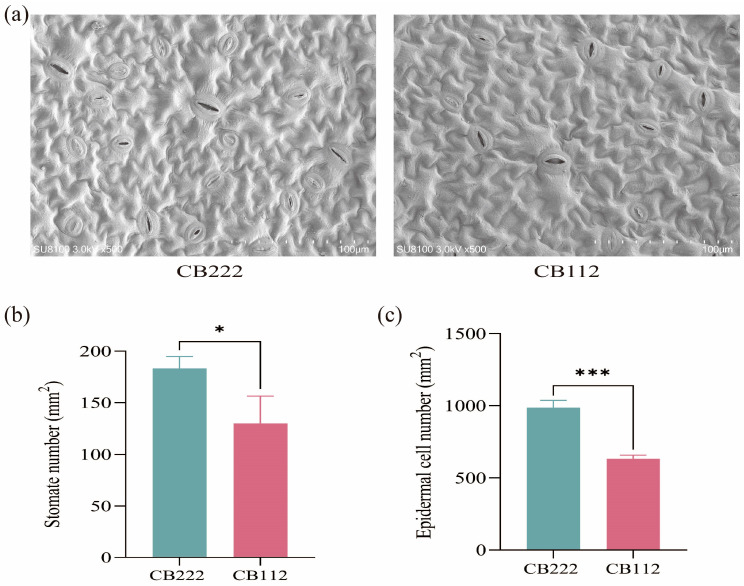
Scanning electron microscopy (SME) observations of CB112 and CB222 leaves. (**a**) CB112 and CB222 leaves; (**b**) stomata number per mm^2^ of CB112 and CB222 leaves; (**c**) epidermal cell number per mm^2^ of CB112 and CB222 leaves. (* denotes *p* < 0.05, *** denotes *p* < 0.001.)

Scanning electron microscopy observations of mature pepper leaves revealed the number of stomata per mm^2^ and the number of epidermal cells per mm^2^ of CB112 and CB222 differed significantly (Figure 2a–c).

### 3.2. Cytological Characterization

**Figure 3:**
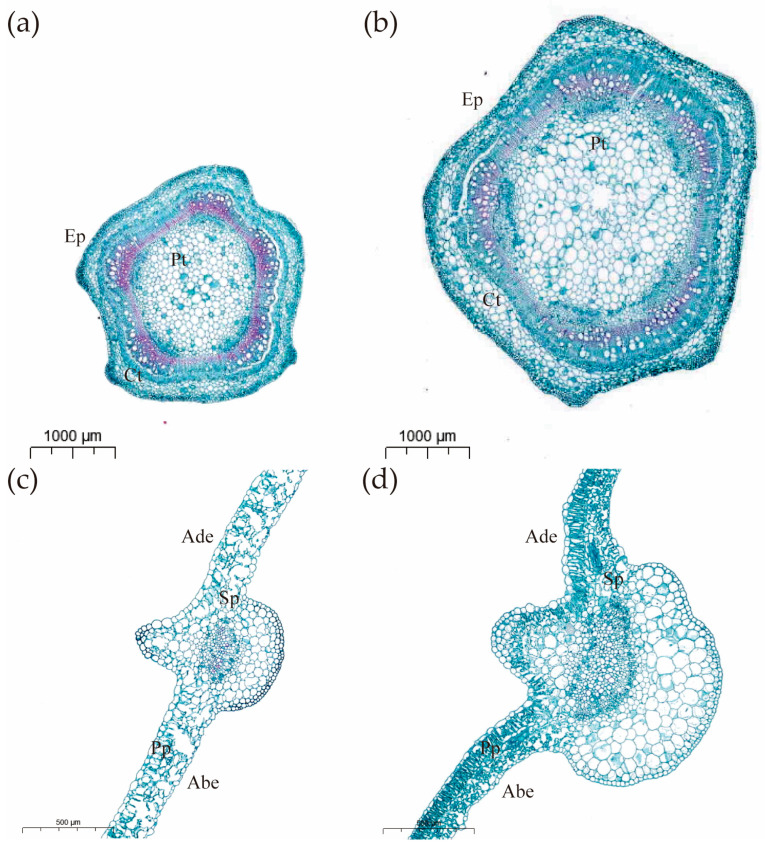
(**a**) CB112 stem cross section (×1.4); (**b**) CB222 stem cross section (×1.4); (**c**) CB112 leaf cross section (×3.5); (**d**) CB222 leaf cross section (×3.5). (Ep: epidermis; Ct: cortex; Pt: pith; Ade: adaxial epidermis; Abe: abaxial epidermis; Sp: spongy parenchyma; Pp: palisade parenchyma.)

The results of the study showed highly significant differences (*p* < 0.01) in leaf thickness, fenestrated tissue cells, epidermal cells, and spongy tissue cells between CB112 and CB222 (Figure 3a,b and Table 1). In stems, stem thickness, epidermal cells, cortex, and pith of CB112 differed highly significantly (*p* < 0.01) from those of CB222 (Figure 3c,d and Table 1).

### 3.3. Differential Accumulation of Phytohormones

**Figure 4:**
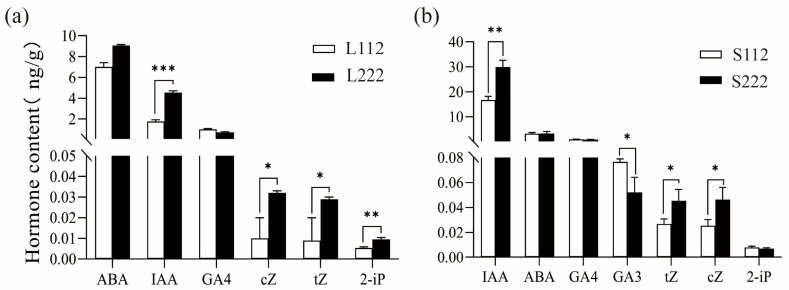
Comparison of the hormone content in CB112 and CB222 pepper plants. (**a**) Differences in hormone content in CB112 and CB222 leaves; (**b**) differences in hormone content in CB112 and CB222 stem internodes. (* denotes *p* < 0.05, ** denotes *p* < 0.01, *** denotes *p* < 0.001.)

The results of the qualitative and quantitative hormonal analysis of all the samples obtained are shown in Table 1. A total of eight phytohormone signals, including IAA, ABA, GA4, GA3, cZ, tZ, 2-iP, and BR, were detected (Table 2). The test results were grouped to calculate the statistical significance of differences. The analysis results showed that IAA and 2-iP were significantly (*p* < 0.01) higher in L222 than in L112 leaves (Figure 4a). Also, cZ and tZ were significantly (*p* < 0.05) higher in L222 than in L112 leaves. In the stem internodes (Figure 4b), IAA, tZ, and cZ were significantly higher in S222 than in S112, while the content of GA3 was higher in S112.

### 3.4. Metabolome Analysis

To further understand which metabolites were associated with the differences in pepper plant height and leaf size, metabolomic analysis of CB112 and CB222 pepper plants were performed using untargeted metabolomics (LC-MS/MS). Metabolome analysis detected 207 differential metabolites, with 107 up-regulated and 100 down-regulated (Figure 4b), of which 11 differential metabolites were associated with plant height and leaf size, including ferulaldehyde, trans-cinnamic acid, sinapyl alcohol, abscisic acid, 2′-deoxyadenosine, 4-aminobutyric acid, L-serine, gibberellin, indole-3-acetic acid, allantoin, and O-phospho-L-serine, which were enriched in 29 of the biosyntheses of secondary metabolites, phenylalanine metabolism, phenylpropanoid biosynthesis, carotenoid biosynthesis, plant hormone signal transduction, and purine metabolism metabolic pathways (Table S1).

### 3.5. Transcriptome Analysis to Identify Differentially Expressed Genes Signal Transduction

To further elucidate the genetic factors underlying differences in pepper plant height and leaf size, RNA-seq was performed using the Novaseq-PE150 platform. After filtering the raw data, a total of 75.68 G clean bases were obtained from the 12 samples of transcriptome data of pepper leaves: 127,590,588 clean reads were obtained from group CB112, 129,529,974 clean reads from group CB222, 124,672,406 clean reads from the Df2 group, 122,663,160 clean reads from group Hf2, and the error rate was less than 0.03%, the percentage of Q20 bases was more significant than 97%, the percentage of Q30 bases was greater than 92%, and the GC content was above 41% (Table S2). Based on the range of matches to the reference genome (Table S2), the CB222-2 sample was excluded, and the CB222-1 and CB222-3 samples were selected as the two biological replicates of the CB222 group for the differential expression gene screen.

**Figure 5:**
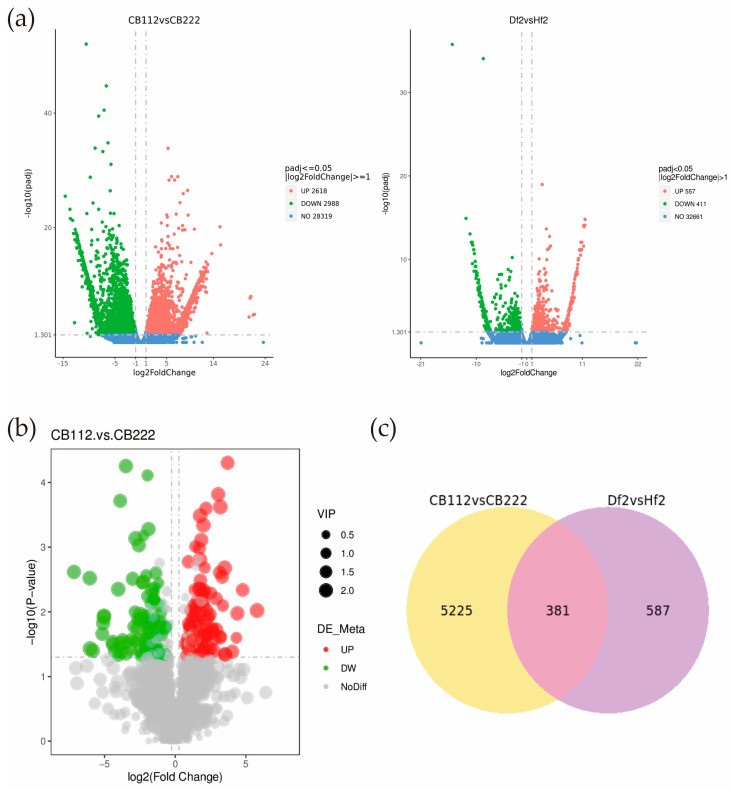
Volcan map of differential metabolites and volcan map and Venn diagram of DEGs. (**a**) CB112 vs. CB222 and Df2 vs. Hf2 volcan map diagram of DEGs; (**b**) common DEGs between CB112 vs. CB222 group and Df2 vs. Hf2 group; (**c**) CB112 vs. CB222 and Df2 vs. Hf2 Venn diagram of DEGs.

Differential expression analysis provided 5606 differentially expressed genes in the CB112 and CB222 groups, including 2618 up-regulated genes and 2988 down-regulated genes (Table S3). A total of 968 differentially expressed genes, including 557 up-regulated genes and 411 down-regulated genes, were obtained in the Df2 and Hf2 groups (Table S4). The intersection of the differentially expressed genes screened in each CB112 and CB222 group and the Df2 and Hf2 groups was taken, and 381 differentially expressed genes were obtained (Figure 5c).

### 3.6. Transcription Expression Analysis

Among the DEGs in the CB112 and CB222 groups, 306 transcription factors were found to be differentially expressed, and they were from different families, including B3, NAC, PLATZ, bHLH, MYB, AP2/ERF, and TCP, among others (Table S5). The B3 gene family is plant-specific, with 16 B3 transcription factors differentially expressed. NAC transcription factors have critical regulatory roles in plant growth and development, biotic and abiotic stress responses, and 16 NAC transcription factors were found to differ. The PLATZ family of transcription factors is a group of plant-specific zinc-dependent DNA-binding proteins that play an indispensable role in plant growth, development, and stress tolerance [22]. There were differences in four PLATZ transcription factors. The bHLH transcription factors are one of the most prominent families of transcription factors in plants and are involved in mediating various signaling and anabolic pathways. There are 14 differential bHLH transcription factors. MYB transcription factors are a more numerous class of transcription factors in plants, with a total of 30 differential MYB transcription factors. AP2/ERF transcription factors are plant-specific, with three AP2 and seven ERF transcription factors differentially expressed. TCP is a group of plant-specific transcription factors that are closely related to cell proliferation and growth, and six TCP transcription factors were found to be differentially expressed in this study.

Among the 968 differentially expressed genes in the Df2 and Hf2 groups, there were 73 differential transcription factors belonging to 29 gene transcription factor families (Table S6), including AP2/ER, AUX/IAA, bHLH, C2H2, GNAT, MYB, NAC, and WRKY.

The differential genes shared by the CB112 and CB222 groups and Df2 and Hf2 groups were analyzed, and 14 transcription factors were screened, belonging to 10 transcription factor families including WRKY, MYB, and NAC.

### 3.7. Functional Enrichment Analysis of Differentially Expressed Genes

**Figure 6:**
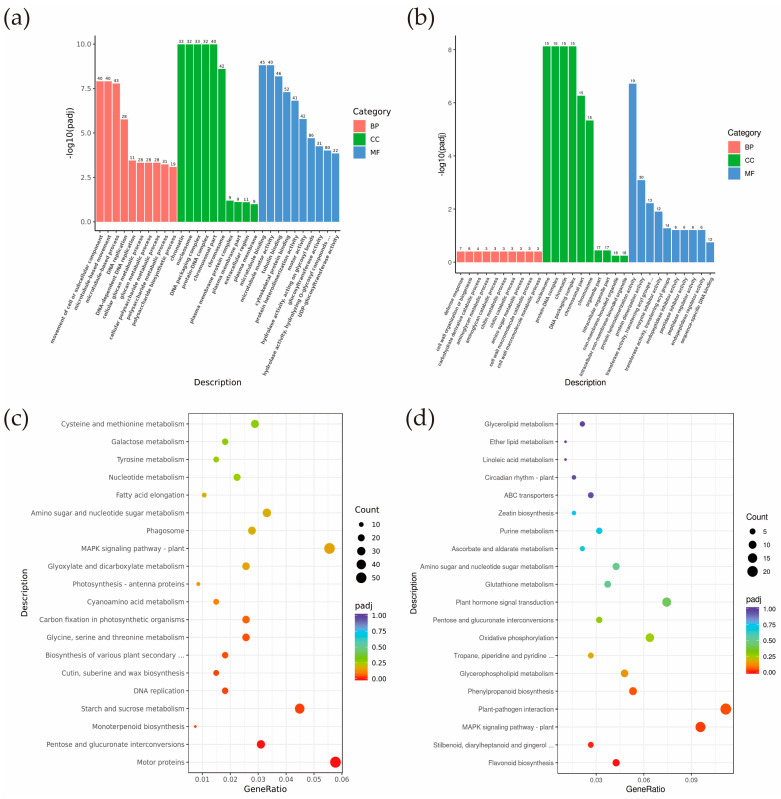
GO and KEGG pathway enrichment analysis. (**a**) CB112 vs. CB222 GO pathway enrichment analysis; (**b**) Df2 vs. Hf2 GO pathway enrichment analysis; (**c**) CB112 vs. CB222 KEGG pathway enrichment analysis; (**d**) Df2 vs. Hf2 KEGG pathway enrichment analysis.

Annotation by GO function revealed that a total of 2043 genes were enriched in 53 GO terms in the CB112 and CB222 groups. Among them, 681 genes were annotated to the Biological Process, 212 genes to the Cellular Component, and 1150 genes to the Molecular Function (Table S7). In BP, it is mainly enriched in the movement of the cell or subcellular component (GO:0006928), microtubule-based movement (GO:0007018), microtubule-based process (GO:0007017), DNA replication (GO:0006260), etc. In CC, it is primarily enriched in the chromatin (GO:0000785), nucleosome (GO:0000786), DNA packaging complex (GO:0044815), etc. In MF, it is mainly enriched in the microtubule binding (GO:0008017), microtubule motor activity (GO:0003777), tubulin binding (GO:0015631), cytoskeletal protein binding (GO:0008092), etc. (Figure 6a).

A total of 164 genes in the Df2 and Hf2 groups received functional annotations, distributed across 10 GO terms. Of these, 90 and 74 genes were annotated in the cellular component and molecular function, with no annotation for the Biological Process (Table S8). It was mainly enriched in the nucleosome (GO:0000786), protein-DNA complex (GO:0032993), chromatin (GO:0000785), etc. in CC. In MF, it was mainly enriched in the protein heterodimerization activity (GO:0046982), protein dimerization activity (GO:0046983), transferase activity, transferring acyl groups other than amino-acyl groups (GO:0016747), and enzyme inhibitor activity (GO:0004857), among others (Figure 6b).

KEGG pathway enrichment analysis was performed for DEGs obtained from the CB112 and CB222 groups and in the Df2 and Hf2 groups from their respective screens. The results showed that DEGs in the CB112 and CB222 groups were significantly enriched in motor proteins (cann04814), pentose and glucuronate interconversions (cann00040), monoterpenoid biosynthesis (cann00902), starch and sucrose metabolism (cann00500), DNA replication (cann03030), cutin, suberine and wax biosynthesis (cann00073), biosynthesis of various plant secondary metabolites (cann00999), and glycine, serine, and threonine metabolism (cann00260) were significantly enriched (Figure 6c). DEGs in the Df2 and Hf2 groups were significantly enriched mainly in flavonoid biosynthesis (cann00941), stilbenoid, diarylheptanoid and gingerol biosynthesis (cann00945), MAPK signaling pathway plant (cann04016), plant–pathogen interaction (cann04626), and phenylpropanoid biosynthesis (cann00940) pathways (Figure 6d). The CB112 and CB222 groups and the Df2 and Hf2 groups were enriched in the MAPK signaling pathway plant (cann04016) and the pentose and glucuronate interconversions (cann00040) pathways.

### 3.8. Functional Enrichment Analysis of Genes Differentially Associated with Plant Height and Leaf Size

**Figure 7:**
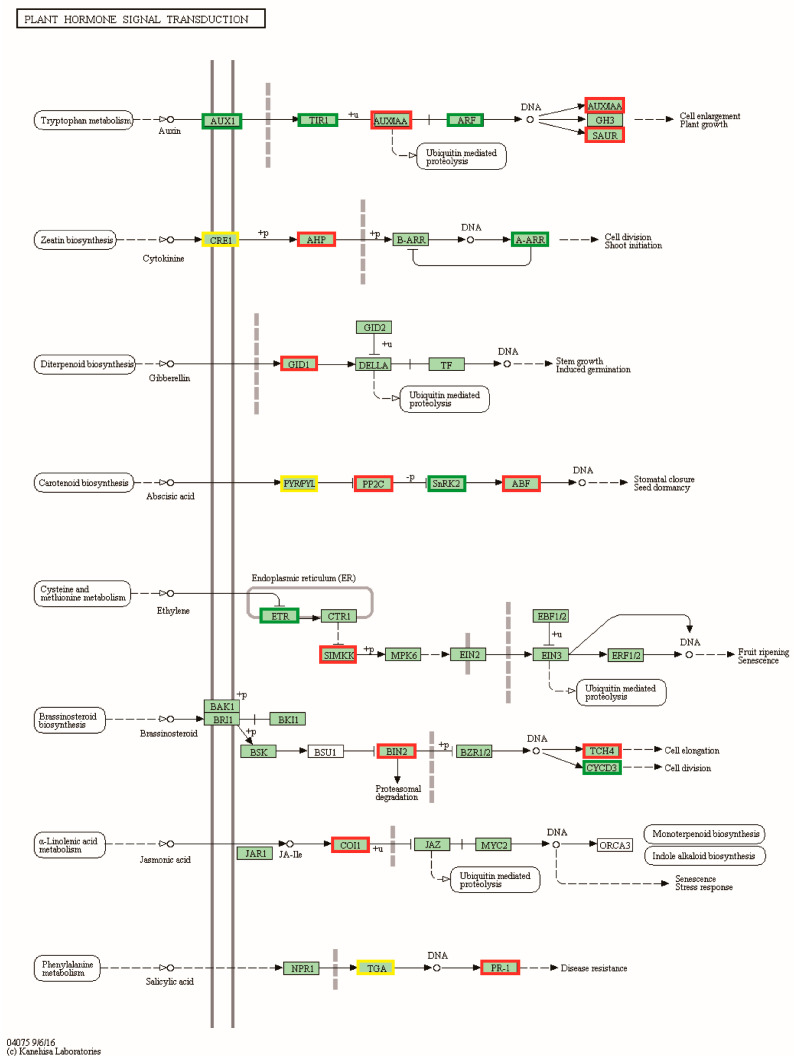
CB112 vs. CB222 plant hormone signal transduction. Red markers are associated with up-regulated genes, green markers with down-regulated genes and yellow markers are associated with both up- and down-regulated genes.

**Figure 8:**
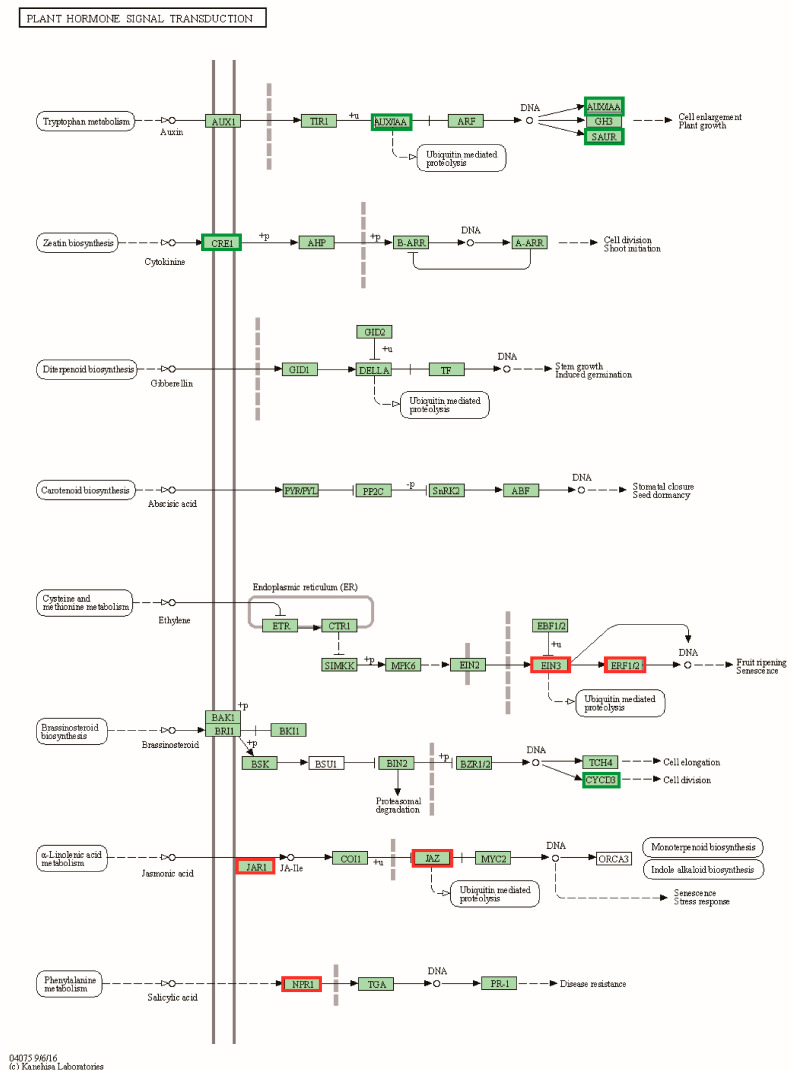
Df2 vs. Hf2 plant hormone signal transduction. Red markers are associated with up-regulated genes and green markers with down-regulated genes.

To further understand the mechanisms associated with pepper plant height and leaf size, our study concentrated on differential genes involved in the plant hormone signal transduction pathway (cann04075) and differential genes shared by both combinations. Thirty-eight differentially expressed genes were identified in the plant hormone signal transduction pathway of the CB112 and CB222 groups (Figure 7). Seven genes exhibited enrichment in the auxin signaling pathway, encompassing auxin-responsive proteins (*SAUR36* and *IAA17*) and auxin response factor (*ARF5*); six genes, including (*AHK3*, *AHK4*, and *ARR15*) were enriched in the cytokinin signaling pathway; *GID1B* was found to be enriched in the gibberellin signaling pathway; and twenty-four genes were enriched in the signaling transduction pathways of the brassinosteroid, abscisic acid, ethylene, and jasmonic acid. Fourteen differentially expressed genes involved in the plant hormone signal transduction pathway were identified in the Df2 and Hf2 combination (Figure 8), including the auxin-responsive protein (*SAUR20*), auxin-responsive promoter (*JAR6*), and auxin-inducible protein (*AUX22*); cyclin-D3-2 (*CYCD3-2*); and ethylene-response factor (*ERF.C.3*) and ethylene insensitive (*EIN3*) genes, etc.

Among the 381 differential genes, gibberellin-regulated protein (*GASA1*), cyclin-D3-2 (*CYCD3-2*), and cytokinin dehydrogenase (*CKX1*) were screened for plant structure growth.

### 3.9. Analysis of Plant Structure Related Differential Genes Using WGCNA

#### 3.9.1. Module Selection

**Figure 9:**
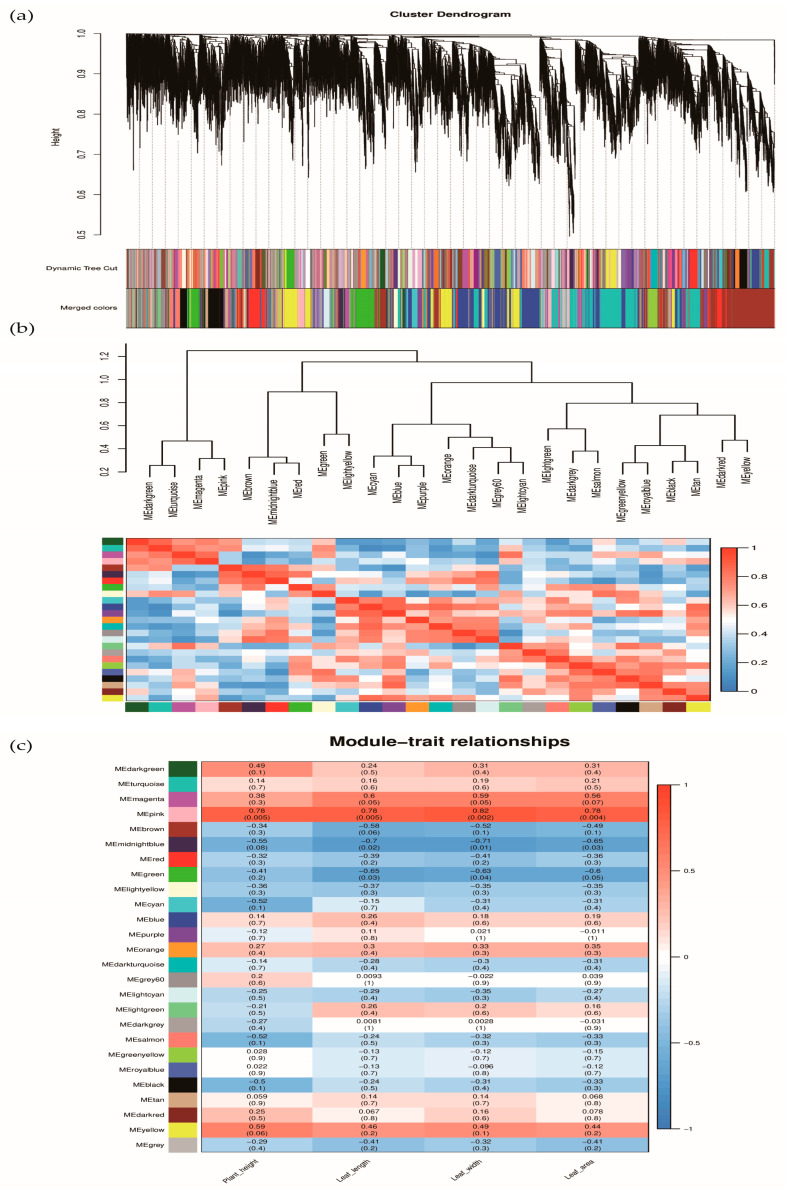
(**a**) Module hierarchy clustering tree diagram; (**b**) Heat map of inter-module correlations; (**c**) Heatmap of association between co-expression network modules of genes and physiological indicators.

RNA-Seq data from 11 pepper leaves were screened and filtered, and 18,800 valid genes were selected for WGCNA analysis. A hierarchical clustering tree was constructed based on the correlation of expression between genes, with each branch of the tree corresponding to a set of genes with highly correlated expressions (Figure 9a). A total of 26 gene co-expression modules were obtained (Figure 9c). Each color represents a module, and grayed-out modules represent genes that could not be assigned to any modules. There were significant differences between the different modules. We analyzed the correlation between the modules and found that the following pairs are more correlated: dark green with the turquoise module, magenta with the pink module, midnight blue with the red module, green with the light-yellow module, blue with the purple module, grey60 with the light cyan module, dark grey with the salmon module, green yellow with the royal blue module, black with the tan module, and dark red with the yellow module. The similarity in the genes between these pairs of modules is stronger (Figure 9b).

The correlation analysis was conducted between 26 gene co-expression modules and plant height and leaf size. The results showed that the co-expression modules significantly associated with the development of pepper plant height and leaf size were the same. Among them, the MEpink module was suggestively and positively correlated with plant height and leaf size traits (r = 0.78, *p* = 0.005; r = 0.78, *p* = 0.002; r = 0.82, *p* = 0.002; r = 0.78, *p* = 0.004).

#### 3.9.2. Screening for Differential Genes Associated with Plant Height and Leaf Size

**Figure 10:**
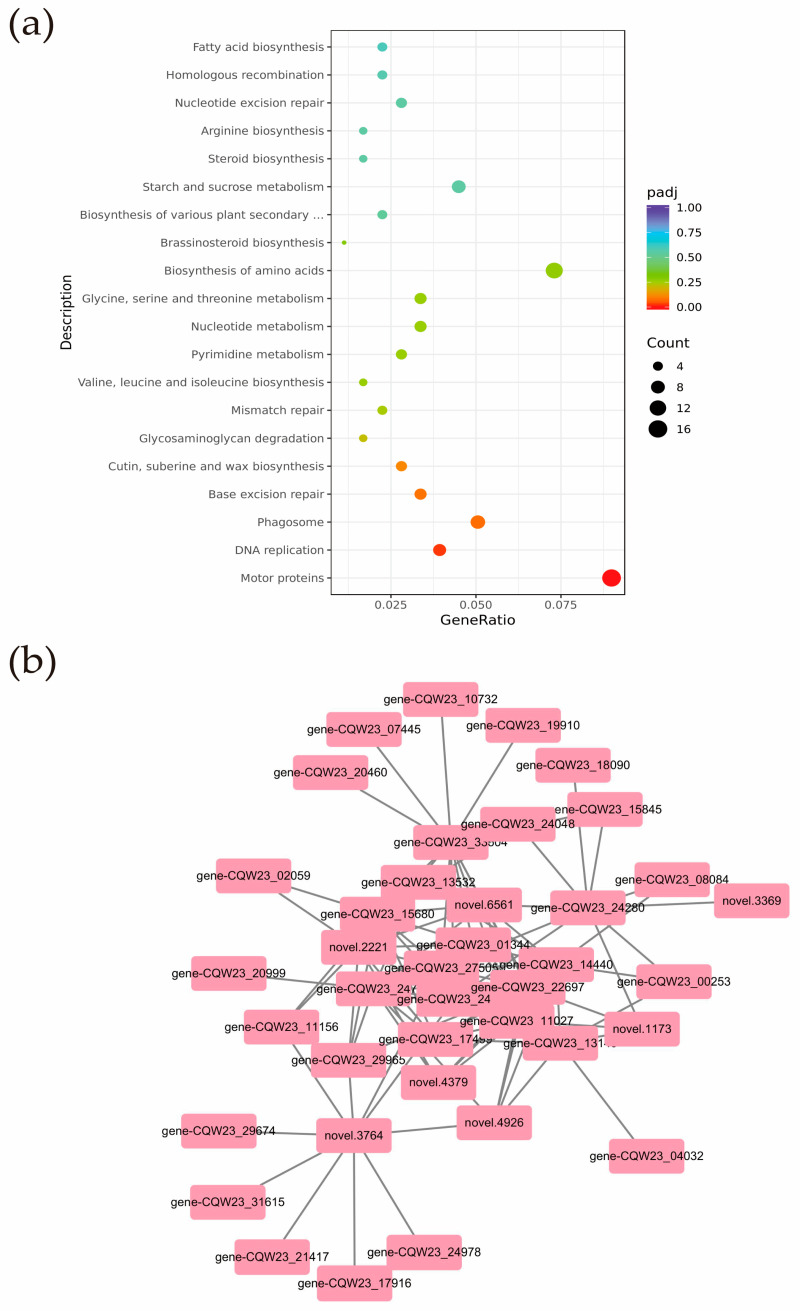
(**a**) KEGG pathways enrichment results of genes in the MEpink module; (**b**) pink module genes correlation network.

A total of 831 genes from the MEpink module were selected for KEGG analysis (Table S9). The results showed that genes in the MEpink module were mainly enriched in pathways such as motor proteins and DNA replication, in which the brassinosteroid biosynthesis pathway was related to phytohormone biosynthesis (Figure 10a). Motor proteins depend on cytoskeletal proteins to convert chemical energy into mechanical energy and are essential for plant and animal cell growth and cell division. Scientists identified and demonstrated a kinesin-type motor protein with transcription factor activity whose mutation resulted in reduced levels of gibberellin (GA) synthesis and blocked cell elongation [23].

The stronger connectivity of a gene within a module indicates a central position. Connectivity is generally expressed as a k-value, and genes with a high k-value can be considered hub core genes. The top 10 genes were screened as central genes based on their connectivity within the pink module. Among the first 10 major genes we found *CCN2* (*CQW23_24704*), *CDC2D* (*CQW23_11027*), *TUBB2* (*CQW23_24280*), *KN* (*CQW23_14440*), and *ROPGAP2* (*CQW23_15680*), for a total of 5 protein genes. These proteins act as central genes that may regulate pepper plant height and leaf size.

To further analyze the genes involved in regulating pepper plant height and leaf size, we constructed a correlation network analysis using the top 10 genes corresponding to the WEIGHT values of each gene in the central gene and network node relationships of the PINK module (Table S10). The results revealed that 39 genes in the pink module were highly correlated with plant height and leaf size (edge weight ≥ 0.215). In addition to the hub genes, 10 protein genes, such as *KIN10A* (*novel.6561*), *CYCA3-1* (*CQW23_11156*), and *TPX2* (*CQW23_17499*), also had a strong relationship with plant height and leaf size regulation (Figure 10b).

### 3.10. Validation Using qRT-PCR

**Figure 11:**
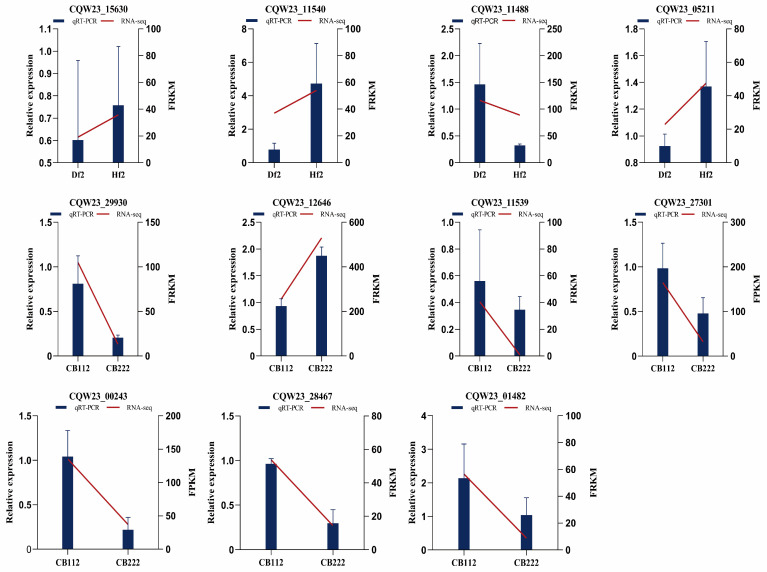
Validation of RNA-seq by qRT-PCR. The histogram data indicate qRT-PCR data (left Y-axis), dashed line indicates RNA-seq data (right Y-axis).

To validate the RNA-seq results, 11 genes with different expression levels and functions were selected for qRT-PCR including the auxin-induced protein (*CQW23_11540*), protein TIFY 10b (*CQW23_11488*), cyclin-D3-2 (*CQW23_05211*), ethylene-responsive transcription factor (*CQW23_29930*), auxin-responsive protein (*CQW23_11539*), abscisic acid receptor (*CQW23_00243*), bZIP transcription factor (*CQW23_28467*), etc. The expression profiles of the 11 genes shown in the results are consistent with the RNA-seq expression results, suggesting the accuracy and reliability of the transcriptome data (Figure 11).

## 4. Discussion

Peppers are a vegetable crop with significant economic value and the traits associated with plant growth like plant height and leaf size directly linked with the photosynthesis process, as well, the plant’s resistance mechanism to stresses directly affected productivity. The structure of a pepper plant depends on the number of primary stem nodes and internode length. The plant stops growing when the main stem reaches its physiological height, as auxin is synthesized in the apical stem tip, inflorescence meristem replaces the function of the apical meristem, and the plant transitions from vegetative growth to reproductive growth [24,25]. Dwarf breeding is an important strategy for breeding wheat, rice, maize, and other crops [26,27,28,29]. Since the 1960s, breeders have triggered the first Green Revolution through the selection and breeding of semi-dwarf varieties that have greatly improved crop resistance and biological yields [30,31,32]. As an organ of plant photosynthesis, the leaf is a trophic organ with remarkable plasticity during plant evolution. Genetic factors control the morphological construction of leaf size, and transcription factors play an essential role in maintaining leaf size.

In this study, two pepper cultivar germplasms, CB112 and CB222, with different plant height and leaf size characteristics were selected to provide a foundation for analyzing the molecular mechanisms underpinning these differences. Differential metabolite identification of pepper plant height and leaf size was performed by untargeted metabolomics and 207 differential metabolites were detected, with 11 differential metabolites related to plant height and leaf size. Among them, gibberellin is a diterpenoid phytohormone that has important roles in plant development, such as stem elongation, hypocotyl elongation, seed development, and promotion of cell division and elongation [33,34,35].

In transcriptomics, after KEGG enrichment analysis, we found the *ERECTA* gene in the MAPK signaling pathway plants (cann04016) in the CB112 and CB222 groups. The gene *ERECTA* was cloned from Arabidopsis [36]. This gene is involved in regulating leaf morphology, stomatal development, inflorescence architecture, and disease resistance. QTL localization indicated that the *ERECTA* gene regulates the number and size of leaf epidermal cells. This regulation ultimately leads to a negative correlation between the number of leaf epidermal cells and cell size and a positive correlation between cell area and the number of rosette leaves [37]. The overexpression of *ZmERECTA* in maize has been demonstrated to enhance plant organ size and yield, as well as to improve transpiration efficiency and increase drought tolerance in maize [38].

A total of 379 differential transcription factors were identified in this study. In particular, 306 differential transcription factors were identified in the CB112 and CB222 groups, including 54 types of B3, NAC, GRF, MYB, TCP, and WRKY, and 73 differential transcription factors in the Df2 and Hf2 groups, which belonged to a family of 29 gene transcription factors, including AP2/ER, AUX/IAA, bHLH, C2H2, GNAT, MYB, NAC, WRKY, B3, C2C2, EIL, GARP, GRAS, ZIP, KNOX, HMG, HSF, Jumonji, MADS, Others, PHD, PLATZ, Rcd1, RWP, SBP, TCP, Tify, TRAF, and Trihelix. There were 26 differentially transcribed families that overlapped in both groups. Among them, TCP is a class of plant-specific transcription factors involved in regulating plant growth and developmental processes. Research has indicated that TCP transcription factors participate in phytohormone synthesis and signal transduction and regulate various pathways such as cell proliferation, cell differentiation, cell wall development, and plant morphogenesis [39]. We detected five of them, *TCP4*, *TCP18*, *TCP20*, *TCP19*, and *TCP13*. In Arabidopsis, *AtTCP20* can interact with genetic elements of *CYCB1*, thereby exerting regulatory control over cell division and growth [40]. *TCP4* regulates cell division, differentiation, and leaf development [41,42]. Under high-temperature conditions, the Class II *TCPs*, specifically *TCP4*, interact with the promoter of the cell cycle-dependent protein kinase inhibitor gene (*ICK1*), thereby controlling cell division and suppressing leaf area expansion [43]. Growth regulators (*GRFs*) are positive regulators in excess of cell proliferation to differentiation during leaf development, and *TCP4* enables the excess of cell proliferation to differentiation by stimulating Mir396B transcriptional targeting to degrade *GRFs* [44].

WRKY transcription factors are one of the most extensive families of transcription factors in plants, named after the WRKY structural domain of the highly conserved heptapeptide WRKYGQK amino acid sequence consisting of 60 amino acids, which is unique to the N terminus. Previous studies have shown that WRKY transcription factors have critical biological functions in plant growth and development and response to stresses. Deletion mutants of rice *OsWRKY53* exhibit characteristics such as reduced leaf inclination and shorter plant stature [45], whereas the overexpression of *OsWRKY78* stimulates the elongation of rice stems [46]. The overexpression of *GhWRKY15* in cotton results in the elongation of the stem [47]. Apple *MdWRKY9* positively regulates plant dwarf development by suppressing the formation of oleuropein steroid (BR) [48]. From the RNA-Seq data, we detected a total of 19 differentially expressed WRKY transcription factors, and the differences in plant height and leaf size may be related to WRKY transcription factors. However, the role of WRKY in the growth and development of peppers has to be further investigated because there are too many members of the WRKY transcription factor family, and their roles are not the same in different plant species.

The plant hormone auxin is crucial in regulating plant height. The auxin content of CB222 was significantly higher than that of CB112 in the main stem internodes and mature leaf tissues. It can be hypothesized that CB222 is subject to more auxin regulation. Auxin in plants can act directly on cell membranes and intracellular components to regulate essential processes such as cell division, elongation, and differentiation [49]. At the molecular level, AUX/IAA, which is one of the three gene families involved in the early response to auxin, plays an important role in the regulation of auxin. A previous study revealed that the overexpression of *OsIAA1* in rice resulted in a significant reduction in plant structure [50]. The SAUR gene is closely associated with cell expansion, and the overexpression of *SAUR36* in Arabidopsis resulted in hypocotyl elongation [51]. The differential genes within the pathway of plant hormone signal transduction are involved in multiple hormone metabolisms and signaling, as analyzed by KEGG enrichment, from which it is, therefore, possible to explore further which specific differential genes are directly involved in influencing the phenotypic differences between CB112 and CB222.

## 5. Conclusions

This study provides initial comprehensive insights into the development of pepper plant height and leaf size through transcriptome, metabolome, and quantification of phytohormones. Twelve differential metabolites associated with the development of plant height and leaf size, along with several candidate genes related to plant hormone signal transduction pathways and differentially expressed transcription factors such as TCP and WRKY, were identified. It provides a theoretical foundation for exploring the molecular mechanisms of pepper growth and development, which can be followed by further functional validation of the candidate genes.

## Figures and Tables

**Table 1:** Anatomical data of CB112 and CB222.

Tissue	Cell Type	CB112	CB222
Leaf	Blade thickness (μm)	129.91 ± 1.41	548.81 ± 41.15 ***
	Epidermal cell (μm^2^)	1008.65 ± 273.54	1428.53 ± 183.26 **
	Palisade parenchyma (μm^2^)	1417.44 ± 191.98	1856.28 ± 225.26 ***
	Spongy parenchyma (μm^2^)	937.99 ± 157.86	1282.44 ± 289.592 **
Stem	Stem diameter (μm)	2846.96 ± 98.24	4548.31 ± 128.72 **
	Epidermal cell (μm^2^)	603.83 ± 174.54	1185.28 ± 210.93 ***
	Cortex cell (μm^2^)	2236.81 ± 497.13	5113.07 ± 853.37 ***
	Pith (μm^2^)	5103.70 ± 1146.01	15,919.46 ± 2844.34 ***

Note: ** denotes *p* < 0.01, *** denotes *p* < 0.001.

**Table 2:** Hormone concentration (ng/g).

Sample_ID	Group	ABA	GA3	GA4	tZ	IAA	cZ	2-iP	BR
CB112-1	CB112	6.696	N/A	1.000	0.020	1.913	0.020	0.0052	N/A
CB112-2	CB112	6.997	N/A	1.074	0.009	1.642	0.009	0.0059	N/A
CB112-3	CB112	7.408	N/A	0.799	0.009	1.759	0.010	0.0054	N/A
CB222-1	CB222	6.497	N/A	0.768	0.029	4.006	0.032	0.0095	N/A
CB222-2	CB222	9.159	N/A	0.699	00025	4.525	0.027	0.0104	N/A
CB222-3	CB222	9.065	N/A	0.440	0.030	4.713	0.033	0.0078	N/A
S112-1	S112	3.718	0.074	0.894	0.028	15.146	0.022	0.0068	N/A
S112-2	S112	2.937	0.078	1.004	0.022	16.840	0.023	0.0080	N/A
S112-3	S112	3.033	0.078	0.827	0.030	18.075	0.031	0.0088	N/A
S222-1	S222	3.828	0.044	0.808	00044	29.554	0.044	0.0064	N/A
S222-2	S222	3.677	0.066	0.880	0.055	32.738	0.057	0.0063	N/A
S222-3	S222	2.359	0.046	0.789	0.037	27.211	0.038	0.0077	N/A

Note: N/A indicates that the substance was not detected.

## Data Availability

Data are contained within the article and Supplementary Materials.

## Supplementary Materials

The following supporting information can be downloaded at: https://www.mdpi.com/article/10.3390/cells13100827/s1, Table S1: Quantitative results of differential metabolites. Table S2: Quality summary of RNA-Seq 12-sample sequencing data. Table S3: CB112 vs. CB222 DEGs. Table S4: Df2 vs. Hf2 DEGs. Table S5: CB112 vs. CB222 different transcription factors. Table S6: Df2 vs. Hf2 different transcription factors. Table S7: CB112 vs. CB222 GO enrichment analysis results. Table S8: Df2 vs. Hf2 GO enrichment analysis results. Table S9: MEpink Module Genes. Table S10: 100 genes in the pink module. Table S11: Ion pairs for quantitative analysis.
